# Metabolic Profiles Reveal Changes in Wild and Cultivated Soybean Seedling Leaves under Salt Stress

**DOI:** 10.1371/journal.pone.0159622

**Published:** 2016-07-21

**Authors:** Jing Zhang, Dongshuang Yang, Mingxia Li, Lianxuan Shi

**Affiliations:** School of life sciences, Northeast Normal University, Changchun, 130024, China; Institute of Genetics and Developmental Biology, Chinese Academy of Sciences, CHINA

## Abstract

Clarification of the metabolic mechanisms underlying salt stress responses in plants will allow further optimization of crop breeding and cultivation to obtain high yields in saline-alkali land. Here, we characterized 68 differential metabolites of cultivated soybean (*Glycine max*) and wild soybean (*Glycine soja*) under neutral-salt and alkali-salt stresses using gas chromatography-mass spectrometry (GC-MS)-based metabolomics, to reveal the physiological and molecular differences in salt tolerance. According to comparisons of growth parameters under the two kinds of salt stresses, the level of inhibition in wild soybean was lower than in cultivated soybean, especially under alkali-salt stress. Moreover, wild soybean contained significantly higher amounts of phenylalanine, asparagine, citraconic acid, citramalic acid, citric acid and α-ketoglutaric acid under neutral-salt stress, and higher amounts of palmitic acid, lignoceric acid, glucose, citric acid and α-ketoglutaric acid under alkali-salt stress, than cultivated soybean. Further investigations demonstrated that the ability of wild soybean to salt tolerance was mainly based on the synthesis of organic and amino acids, and the more active tricarboxylic acid cycle under neutral-salt stress. In addition, the metabolite profiling analysis suggested that the energy generation from β-oxidation, glycolysis and the citric acid cycle plays important roles under alkali-salt stress. Our results extend the understanding of mechanisms involved in wild soybean salt tolerance and provide an important reference for increasing yields and developing salt-tolerant soybean cultivars.

## Introduction

Cultivated soybean (*Glycine max*) and wild soybean (*Glycine soja*) both belong to *Leguminosae*, *Papilionoideae*, *Glycine*, *Soja*. Cultivated soybean is a very important economic and oil crop, producing 30% of the world's edible oil and 69% of its dietary protein [1]. However, the adaptability of cultivated soybean to adverse environments, especially those under salt stress, was significantly reduced during the process of artificial domestication and cultivation. Additionally, cultivated soybean is a typical glycophyte [2]. Under salt stress, the plant height and leaf area of cultivated soybean decreased, the protein content and the quality of the seeds decreased, and the nitrogen fixation ability was inhibited, thus constraining growth and yield [3, 4]. It is estimated that more than 20% of the world's arable land area was high salt soils [5], and the salinization of soil has become a major threat to agricultural production and distribution worldwide [6]. Additionally, the effects of soil alkalization on crops are more serious than salt stress [7]. To ensure crop yields in saline soil, it is necessary to improve the salt tolerance of soybean cultivars.

Wild soybean is a closely related ancestor of cultivated soybean [8]. It is more suitable to adverse environments than the cultivated soybean, especially under salt stress. Thus, research on the salt tolerance of wild soybean has been increasingly studied, with an emphasis on the mechanism behind its salt tolerance. Xue et al. [9] showed that the Na^+^ level was reduced in the leaves when wild soybean were exposed to salt stress and increased in the roots to avoid excessive Na^+^ at the photosynthetic sites in leaves, and a high level of the photosynthetic activity was maintained to resist external salt damage to the plant. Wild soybean has rapid responses, accumulating a higher level of antioxidant enzymes that reduce the serious damage caused by a large amount of reactive oxygen species (ROS) in plants [10]. Wild soybean could also be adapted to salt stress through the secondary metabolic pathway of isoflavone [11]. Similarly, the expression of *GsNAC20*, a novel NAC transcription gene cloned from wild soybean as an ideal material for cloning resistance genes by Cai et al. [12], was induced by low temperature, salt stress and drought, and also had different response patterns in roots and stems. Presently, the mechanism of salt tolerance in wild soybean was analyzed based on metabolism technology, which revealed that wild soybean contained greater amounts of acetylated amino acids, disaccharides and sugar alcohols than cultivated soybean, but had lower amounts of unsaturated fatty acids, carboxylic acids and monosaccharides [8].

Metabolic phenotype analysis is a neoteric and promising methodology for genomic studies. Metabolic phenotypes and a direct correlation with genotypes were tested using plant metabolomics under certain conditions [13]. The accumulation of plant metabolites and the secondary metabolism are not only closely related to plant growth, but are also regulated by other factors in the environment. Thus, metabolomics reveals the connection between plant and environment, which is accomplished through a thorough understanding of the relationships among function, metabolic networks, metabolic regulation, phenotype and plant growth [14, 15]. The technologies of gas chromatography-mass spectrometry (GC-MS), nuclear magnetic resonance (NMR) and liquid chromatography-mass spectrometry (LC-MS) have been widely used in the analysis of the metabolism, allowing for significant progress in metabolomics in plant sciences. Previous studies revealed metabolic changes under different stresses based on metabolomics in tobacco [16], barley [17], maize [18], wheat [19], tomato [20], *Arabidopsis* [21, 22] and other test materials. They provided new ideas on plant stress physiology, especially the physiological mechanism of salt tolerance. Currently, there are few studies comparing wild soybean and cultivated soybean [8]. As far as we know, this is the first metabolomics-based report revealing different metabolic responses in wild soybean and cultivated soybean at the same latitude under neutral- and alkali-salt stresses.

In the present study, we characterized the metabolic changes of wild soybean and cultivated soybean belonging to *Soja* as experimental materials under artificial simulations of neutral-salt and alkali-salt stresses using GC−MS, to analyze the metabolomic changes in two kinds of *soja* plants under salt stress. The primary objectives of this work are to investigate differences in growth and metabolic profiles between wild soybean and cultivated soybean under different types of salt stress, and to investigate the changes in metabolism plasticity in cultivated soybean under salt stress through its long-term artificial selection from wild soybean. This study provides a theoretical basis for the excellent gene mining of wild soybean, the genetic basis for broadening soybean cultivars and the sustainable production of soybean, and also provides a quantitative parameter system for the cultivation of soybean. Meanwhile, it also has significance as a reference for the study of plant evolution.

## Materials and Methods

### Plant materials and sand cultures

In this experiment, we selected cultivated soybean (M, jinong24) and common wild soybean (W, Huinan06116) at the same latitude in the northeast of China as the experimental materials. Soybean seeds were kindly provided by the New Crop Breeding Center of Jilin Province, China. The soybean seedlings were sand cultured. The river sand, cleaned and sieved, was arranged in 14 cm diameter pots with a bottom hole (2 cm in diameter). Healthy and uniform M and W seeds were selected, and the clay membranes of the W seeds were cut with a knife in advance. Then, the seeds were sown in pots, with three seeds of a single strain per pot, and grown side-by-side in an outdoor area at the Experimental Field of Northeast Normal University, Changchun, Jilin, from May to June of 2015. During this experiment, the temperatures were 18.5±1.5°C at night and 26±2°C in the day time and the humidity was 60%±5%.

### Plant growth conditions and salt stress treatments

After seedling emergence, each pot was watered once with 1× Hoagland nutrient solution every morning (6:00–7:00 AM). Soybeans were treated when they grew third leaves. Before treatment, four pots were used to measure the basic biomasses of each genotype of soybean. In the salt-treated group, the two soybean genotypes were exposed to neutral-salt stress (NaCl and Na_2_SO_4_, at a 1:1 molar ratio, 45 mmol·L^-1^ Na^+^) and alkali-salt stress (Na_2_CO_3_ and NaHCO_3_, at a 1:1 molar ratio, 45 mmol·L^-1^ Na^+^). These seedlings were treated with two types of stress solutions containing 15 mmol·L^-1^ Na^+^ for the first two days, and then treated with 30 mmol·L^-1^ Na^+^ for the next two days, to allow seedlings to gradually adapt to the two types of stress. Finally, W and M were exposed to the two treatments having 45 mmol·L^-1^ Na^+^ for 14 days. In the control group, soybeans were cultivated under normal conditions (1× Hoagland solution). Additionally, 8 pots were set up as a duplicate experiment, resulting in 56 pots included the 8 pots used to measure the basic biomass. Data were recorded and images of the plant's growth status were taken every day. After two weeks, four biological replicates from each treatment of the soybean genotypes were selected randomly as test materials, and the third leaf from the top was harvested. Then, samples were immediately frozen in liquid nitrogen and stored at -80°C to extract metabolites. The other four biological replicates were used to measure growth parameters. The values of shoot height, root length, and dry weight (DW) of shoots and roots were measured according to Shi et al. [23], and the relative growth rates (RGRs) of shoots and roots were determined according to Kingsbury et al. [24] as follows: RGR = (In DW_1_ -In DW_0_) / t_2_ -t_1_.

### Metabolite extraction and metabolite profiling analysis

Approximately 100 mg of each frozen soybean leaf tissue was transferred into 2 ml centrifuge tubes, then 60 μl of water containing ribitol was added to each tube as an internal standard. The mixtures were vortexed. Then, 0.3 ml of methanol and 0.1 ml of chloroform were added. After mixtures were vortexed, a 70 Hz grinding mill system (Jinxin Biotech LTD. Shanghai, China) was used to grind the samples for 5 min, followed by an incubation at 70°C for 10 min. Subsequently, the tubes were centrifuged at 12,000 rpm at 4°C for 10 min. Then, 0.35 ml of supernatant was decanted into a 2-ml volume screw-top glass tube, and samples were dried in a vacuum concentrator at 30°C for 2 h. Afterward, each sample was dissolved in 80 μl of methoxamine hydrochloride (20 mg/ml in pyridine) and incubating at 37°C for 2 h. Samples were further derivatized with N,O-bis (trimethylsilyl)-trifluoroacetamid (BSTFA) containing 1% trimethylchlorosilane (TMCS) (100 μl) at 70°C for 1 h. When the samples cooled to room temperature, the GC-MS analysis was performed using a one-dimensional Agilent 7890 gas chromatograph system coupled with a Pegasus 4D time-of-flight mass spectrometer. A 1 μl aliquot of the analyte was injected in splitless mode. Helium was used as the carrier gas, the front inlet purge flow was 3 ml·min^−1^, and the gas flow rate through the column was 1 mL·min^−1^. The initial temperature was kept at 90°C for 0.25 min, then raised to 180°C at a rate of 10°C·min^−1^, then raised to 240°C at a rate of 5°C·min^−1^, and finally to 285°C at a rate of 20°C·min^−1^ for 11.5 min. The injection, transfer line, and ion source temperatures were 280, 270 and 220°C, respectively. The energy was -70 eV in electron impact mode. The mass spectrometry data were acquired in full-scan mode with the m/z range of 20–600 at a rate of 100 spectra per second after a solvent delay of 492s.

### Data processing and multivariate data analysis

The data was acquired and pre-processed using the manufacturer’s ChromaTOF software (versions 2.12, 2.22, 3.34; LECO, St. Joseph, MI, USA) [25]. Metabolites were identified by searching the commercial EI-MS library, FiehnLib [26]. Afterwards, features with at least an 80% missing value were removed. In the following, the missing value was filled using a small value that was half of the minimum positive value in the original data. And then, the data were filtered by interquantile range (IQR). Then, the total mass of the signal integration area was normalized for each sample. Next, a principal component analysis (PCA), partial least squares discriminant analysis (PLS-DA), orthogonal partial least squares discriminant analysis (OPLS-DA) and loading plot were performed by SIMCA-P 13.0 software package (Umetrics, Umea, Sweden) using normalized data. And variable importance values (VIP) were obtained through PLS-DA and OPLS-DA analyses. In addition, differential metabolites were found using Student’s *t* test (P<0.05) and VIP (VIP>1) combined with similarity values greater than 700. Subsequently, a metabolic pathway was constructed according to KEGG (http://www.genome.jp/kegg/) and a pathway analysis on the MetaboAnalyst website (www.metaboanalyst.ca/) based on the changes in metabolite concentrations compared with those of the respective controls [27].

## Results

### Growth performance

**Fig 1:**
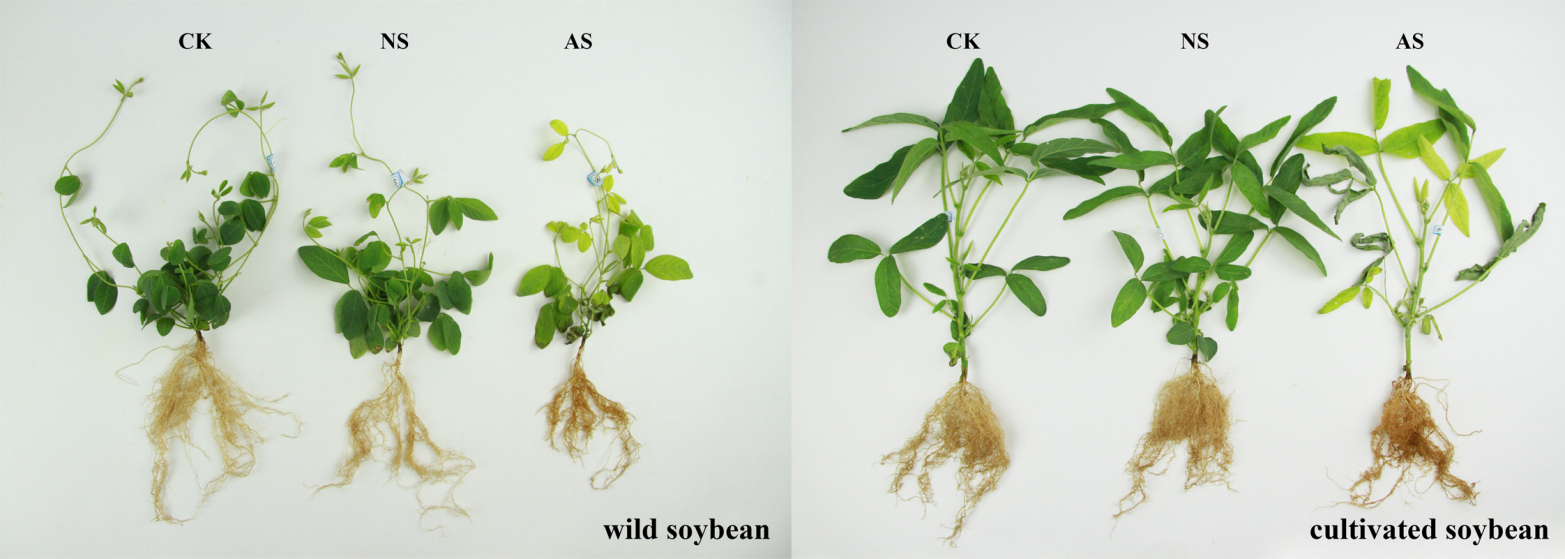
The growth performances of the two soybean genotypes under normal and two salt conditions. CK: control treatment, NS: neutral-salt stress, AS: alkali-salt stress.

Differences in growth performances were observed between W and M under neutral-salt and alkali-salt stresses, containing 45 mmol^-1^ Na^+^ for 14 days after the imposition of treatments, compared with controls (Fig 1). The leaf area and the number of whole plants leaves were reduced under salt stress (Fig 1). The lengths of roots extended into the sand, and the amounts of lateral and fibrous roots were negatively affected by salt stress. Additionally, the root color deepened after salt stress (Fig 1). Compared with the control, regions of the shoots and roots had obvious changes, including a decrease in shoot height, root length, DW of shoots, DW of roots, RGR of shoots and RGR of roots, after both types of salt stresses S1 Table. Alkali-salt stress caused greater decreases in growth parameters in both genotypes compared with neutral-salt stress. In addition, the resistance ability of W to salt stress was greater than M (Fig 1 and S1 Table.

### Metabolite profiles

**Fig 2:**
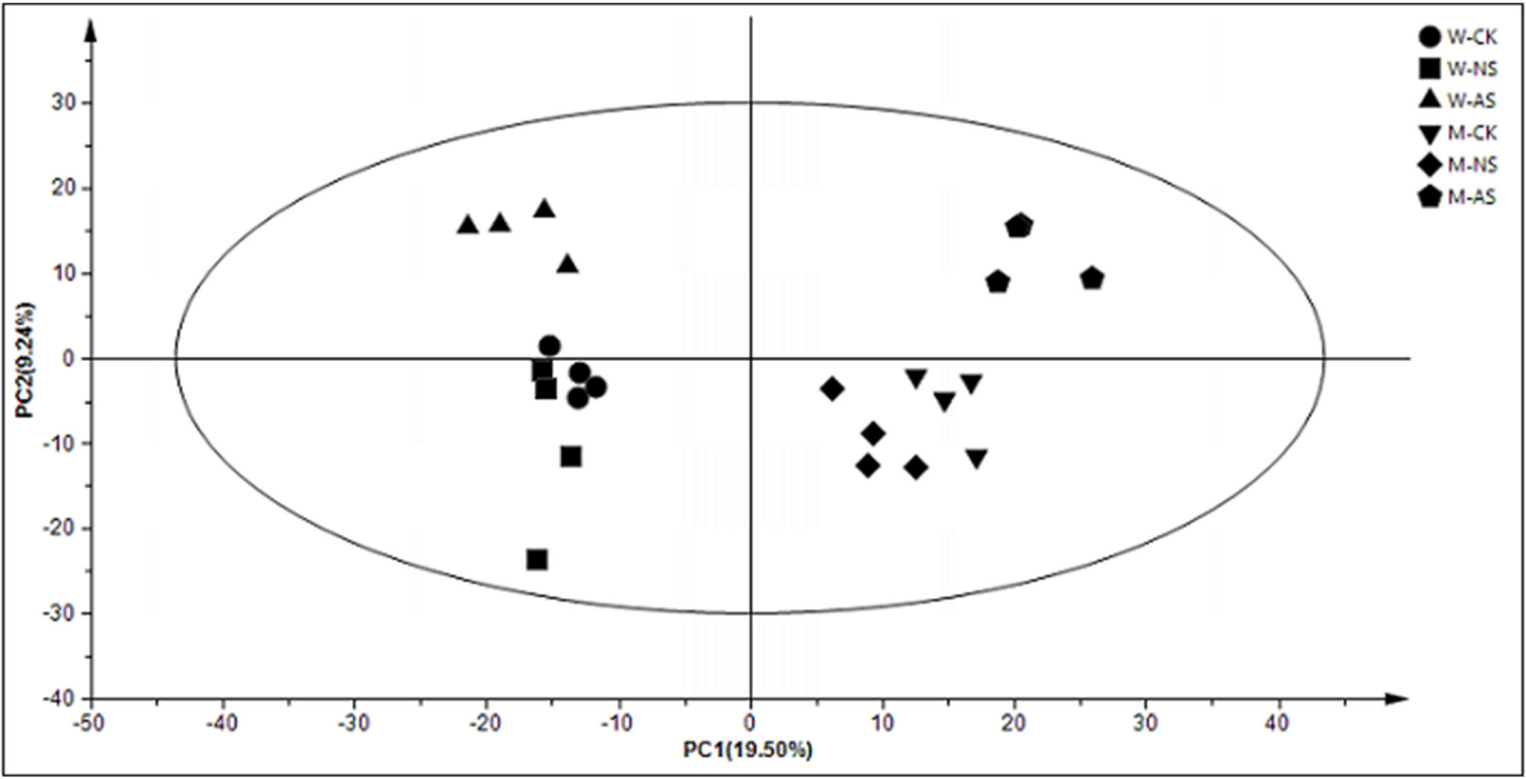
Principal component analysis (PCA) of metabolic profiles in the leaves of W and M under control and 45 mmol.L^-1^ neutral-salt and alkali-salt stress (four biological replicates). W: wild soybean, M: cultivated soybean; CK: control treatment, NS: neutral-salt stress, AS: alkali-salt stress; PC1, the first principal component; PC2, the second principal component.

**Fig 3:**
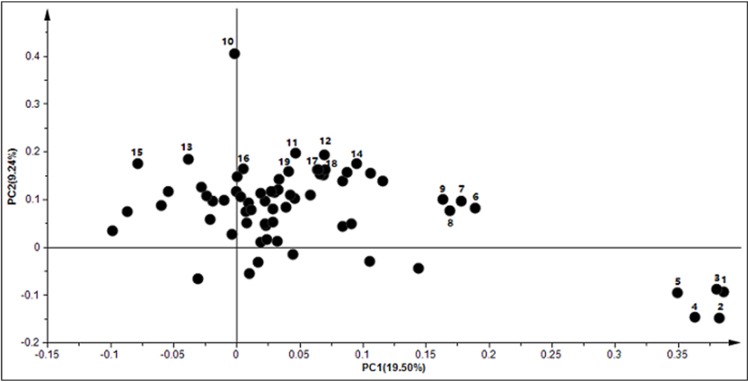
The loading plot of metabolites to the PC1 and PC2. Number 1–9 indicate the metabolites are contributed to PC1, and numbers 10–19 indicate a large contribution rate of metabolites for PC2. 1: aspartic acid; 2: asparagine; 3: phenylalanine; 4: 3-cyanoalanine; 5: glutamic acid; 6: threonine; 7: proline; 8: valine; 9: tyrosine; 10: raffinose; 11: ribose; 12: fumaric acid; 13: pyruvic acid; 14: threonic acid; 15: maltose; 16: phytol; 17: ferulic acid; 18: galactonic acid; 19: glucose. PC1: the first principal component; PC2: the second principal component.

The retention time of W and soybean, as well as controls, under both salt treatments were stable and reproducible, revealing the dependability of metabolite detection and the metabolomic analysis. Differential metabolites were discussed by comparing W to M in seedling leaves responding to neutral- and alkali-salt stresses. A total of 68 compounds (P<0.05, VIP>1 and similarity value>700), including 12 amino acids, 27 organic acids, 11 carbohydrates and polyols, 10 fatty acids and 8 other compounds, were quantified and identified in each polar extract (S2 Table). Hence, the PCA could directly reflect the development of a visual plot for the evaluation of the differences and consistencies in the metabolite profiles of W and M on the basis of the differential metabolites (Fig 2). The PCA showed that there was an obvious separation in genotypes and treatments. The genotypes, W and M, were clearly separated by PC1, which explained 19.50% of the variance. PC2 distinguished the samples from the control and two types of salt treatments, represented 9.24% (Fig 2). Some amino acids, including aspartic acid, asparagine, phenylalanine, glutamic acid, threonine, valine and tyrosine, were major contributors to PC1, while the contribution of metabolites to PC2 was comprised of some organic acids and carbohydrates, including fumaric acid, pyruvic acid, threonic acid, ferulic acid, galactonic acid, raffinose, ribose, maltose and glucose (Fig 3 and S3 Table).

### Metabolic differences under normal condition

To research the metabolic changes between M and W, we compared the metabolic profile of M with that of W under normal condition. We focused on the total metabolites identified, and 29 metabolites, including 2 amino acids, 13 organic acids, 4 carbohydrates and polyols, 7 fatty acids and 3 other compounds, had greater content levels in W than in M (S4 Table). In addition, 39 metabolites, including 10 amino acids, 14 organic acids, 7 carbohydrates and polyols, 3 fatty acids and 5 other compounds, had greater accumulation levels in M more than in W (S4 Table). A further comparative analysis indicated the largest difference between the two genotypes and that the metabolite contents were obviously different between W and M (S4 Table). The metabolite levels that were higher in W than in M consisted of mainly organic acids, including citric acid, proline, citraconic acid, mucic acid, galactonic acid, fumaric acid, dehydroascorbic acid, 4-aminobutyric acid and ferulic acid. The largest difference between W and M concerned the citric acid content, with W having a higher level, indicating that W may have a higher citric acid cycle capability and generate more energy. In contrast, M had higher amino acid and organic acid levels, including lacticacid, oxoproline, citramalic acid, α-ketoglutaric acid, pyruvic acid, D-glyceric acid, D-glycerol-1-phosphate, succinic acid, L-malic acid, salicylic acid, threonic acid and serine, isoleucine, glutamic acid, alanine, glycine, β-alanine, phenylalanine, valine, threonine and tyrosine, in seedling leaves (S4 Table).

### Metabolic profiles in response to neutral-salt stress

**Fig 4:**
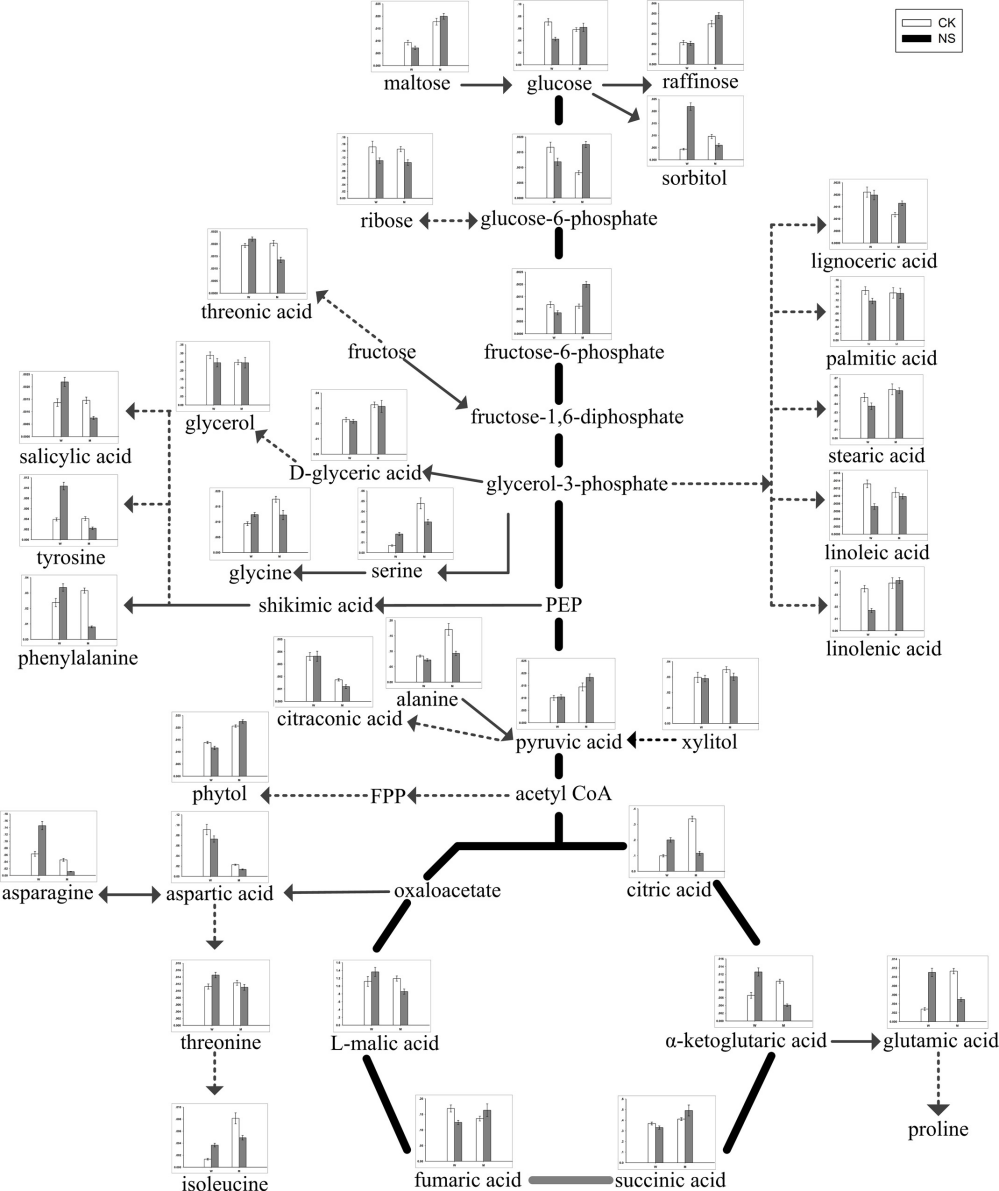
Changes in metabolites of the metabolic pathways in the leaves of the two soybean genotypes seedlings varied with salt tolerance 14 days after the imposition of neutral-salt stress. W and M on the X-axis indicate wild soybean and cultivated soybean, respectively. The values on the Y-axis indicate the relative concentration of metabolites. CK: control treatment, NS: neutral-salt stress.

According to the PCA and the screened differential metabolites, changes in metabolites varied with different salt treatments and genotypes (Fig 2 and Table 1). For W and M, there were 40 metabolites, including 6 fatty acids, 7 carbohydrates and polyols, 8 organic acids, 10 amino acids and 9 metabolites associated with glycolysis and TCA, that showed dramatic changes under neutral-salt stress (Table 1). In the two genotypes, some metabolites responded similarly under the neutral-salt stress. The contents of fatty acids, including glycerol, linolenic acid, linoleic acid, stearic acid, palmitic acid and lignoceric acid in W, and glycerol, linoleic acid, stearic acid and palmitic acid in M, showed decreasing trends. Additionally, the level of some carbohydrates and polyols, including ribose and xylitol, were reduced under neutral-salt stress (Table 1). More importantly, the responses to neutral-salt stress in W and M were different. The accumulation of some organic acids, including threonic acid, citraconic acid, salicylic acid and citramalic acid, were enhanced in W and reduced in M (Table 1). Moreover, the majority of the amino acids were accumulated significantly in W. These amino acids included glutamic acid, tyrosine and isoleucine. In addition, the contents of most of the amino acids in M were reduced, and glycine, aspartic acid, asparagine and tyrosine were significantly reduced (Table 1). The contents of glucose, glucose-6-phosphate and fructose 6-phosphate, which are intermediate metabolites of glycolysis, were reduced in W and increased in M. Although pyruvate is the end product of glycolysis, and both accumulate in W and M, the level of accumulation in M is more than in W. Furthermore, citric acid, α-ketoglutarate and L-malic acid which were intermediates of the TCA cycle associated with glycolysis pathway, accumulated in W, indicating that the TCA process was increased, which resulted in more energy [17]. However, the responses of citric acid, α-ketoglutarate and L-malic acid were inversed in M when induced by neutral-salt stress (Fig 4 and Table 1). The biosynthetic and degradation routes of differential metabolites under the neutral-salt stress of W and M seedlings are presented in Fig 4.

**Table 1:** Relative concentrations and fold changes of differential metabolites in the leaves of W and M after 14 days of neutral-salt stress.

Metabolite name	Relative concentration				Fold changes	
	W		M		log_2_^(NS/CK)^	
	CK	NS	CK	NS	W	M
**Glycolysis and TCA**						
glucose	0.71±0.05	0.42±0.03	0.58±0.03	0.62±0.07	-7.39*	0.91
glucose-6-phosphate	0.02±0.00	0.01±0.00	0.01±0.00	0.02±0.00	-4.86*	10.77*
fructose-6-phosphate	0.01±0.00	0.01±0.00	0.01±0.00	0.02±0.00	-4.73*	8.42*
pyruvic acid	0.10±0.01	0.10±0.01	0.14±0.02	0.18±0.01	0.47	3.39
citric acid	0.99±0.08	2.00±0.14	3.35±0.18	1.15±0.13	10.20*	-15.45**
α-ketoglutaric acid	0.07±0.01	0.13±0.01	0.10±0.01	0.04±0.00	9.40*	-13.29**
succinic acid	3.69±0.14	3.31±0.15	4.11±0.13	4.92±0.51	-1.58*	2.60*
fumaric acid	1.69±0.11	1.24±0.07	1.36±0.08	1.63±0.20	-4.43*	2.58
L-malic acid	11.19±1.24	13.63±1.14	11.93±0.66	8.60±0.66	2.84*	-4.73*
**Amino acids**						
alanine	0.85±0.03	0.72±0.04	1.71±0.19	0.93±0.07	-2.36*	-8.75*
glycine	0.09±0.01	0.12±0.01	0.17±0.01	0.12±0.02	3.92*	-5.07*
serine	0.07±0.01	0.18±0.02	0.48±0.05	0.30±0.02	13.81*	-6.80*
threonine	0.11±0.01	0.15±0.01	0.12±0.01	0.11±0.01	3.74*	-1.60
aspartic acid	0.91±0.10	0.73±0.06	0.23±0.01	0.14±0.01	-3.25*	-7.42*
glutamic acid	0.03±0.00	0.11±0.01	0.11±0.01	0.05±0.00	19.69**	-11.90*
phenylalanine	0.24±0.03	0.34±0.02	0.32±0.02	0.08±0.01	4.95*	-19.66**
asparagine	0.63±0.07	1.46±0.12	0.46±0.04	0.11±0.01	12.06*	-20.42**
tyrosine	0.04±0.00	0.10±0.01	0.04±0.00	0.02±0.00	14.05*	-8.85*
isoleucine	0.01±0.00	0.04±0.00	0.08±0.01	0.05±0.00	14.60*	-7.29*
**Carbohydrates and polyols**						
maltose	0.09±0.01	0.07±0.01	0.18±0.01	0.20±0.01	-3.77	1.68
ribose	1.52±0.17	1.11±0.08	1.45±0.08	1.05±0.08	-4.48*	-4.61*
raffinose	0.02±0.00	0.02±0.00	0.04±0.00	0.05±0.00	-0.49	2.68*
phytol	0.14±0.00	0.12±0.01	0.21±0.00	0.23±0.01	-2.51*	1.27*
xylitol	0.30±0.03	0.29±0.02	0.35±0.02	0.30±0.02	-0.39	-2.06*
sorbitol	0.04±0.00	0.22±0.02	0.10±0.01	0.06±0.01	23.20*	-6.82*
threitol	0.01±0.00	0.01±0.00	0.01±0.00	0.01±0.00	6.07*	-7.78*
**Fatty acids**						
glycerol	2.89±0.19	2.45±0.25	2.48±0.14	2.45±0.31	-2.34*	-0.16
linolenic acid	0.35±0.03	0.17±0.02	0.40±0.04	0.42±0.02	-10.49*	0.73
linoleic acid	0.01±0.00	0.01±0.00	0.01±0.00	0.01±0.00	-8.62*	-1.43
stearic acid	0.48±0.05	0.37±0.04	0.57±0.06	0.55±0.03	-3.49*	-0.39
palmitic acid	1.49±0.11	1.17±0.08	1.42±0.16	1.40±0.16	-3.42*	-0.17
lignoceric acid	0.02±0.00	0.02±0.00	0.01±0.00	0.02±0.00	-0.86	4.85*
**Organic acids**						
threonic acid	0.02±0.00	0.02±0.00	0.02±0.00	0.01±0.00	1.81*	-5.84*
citraconic acid	0.04±0.00	0.04±0.00	0.02±0.00	0.01±0.00	0.06	-5.46**
salicylic acid	0.01±0.00	0.02±0.00	0.01±0.00	0.01±0.00	6.85*	-9.60*
2-ketoadipate	0.01±0.00	0.01±0.00	0.01±0.00	0.00±0.00	1.31	-15.39**
methylmalonic acid	0.56±0.06	0.58±0.05	0.17±0.02	0.12±0.01	0.68	-4.95*
3-cyanoalanine	0.10±0.01	0.26±0.01	0.08±0.00	0.03±0.00	14.07*	-12.83*
citramalic acid	0.01±0.00	0.01±0.00	0.02±0.00	0.01±0.00	1.59*	-7.41**
D-glyceric acid	0.23±0.02	0.21±0.01	0.32±0.02	0.31±0.04	-0.74	-0.48

The relative content of metabolites are the mean of data from four biological replicates using GC-MS. The fold changes was calculated using the formula log_2_^(neutral-salt/control)^. Values were presented as the mean ±standard deviation of four biological replicates. Relative concentration values and standard deviation were increased 10 times in each treatment.

* and **indicate significant (P<0.05) and highly significant difference (P<0.01), respectively.

### Metabolic profiles in response to alkali-salt stress

**Fig 5:**
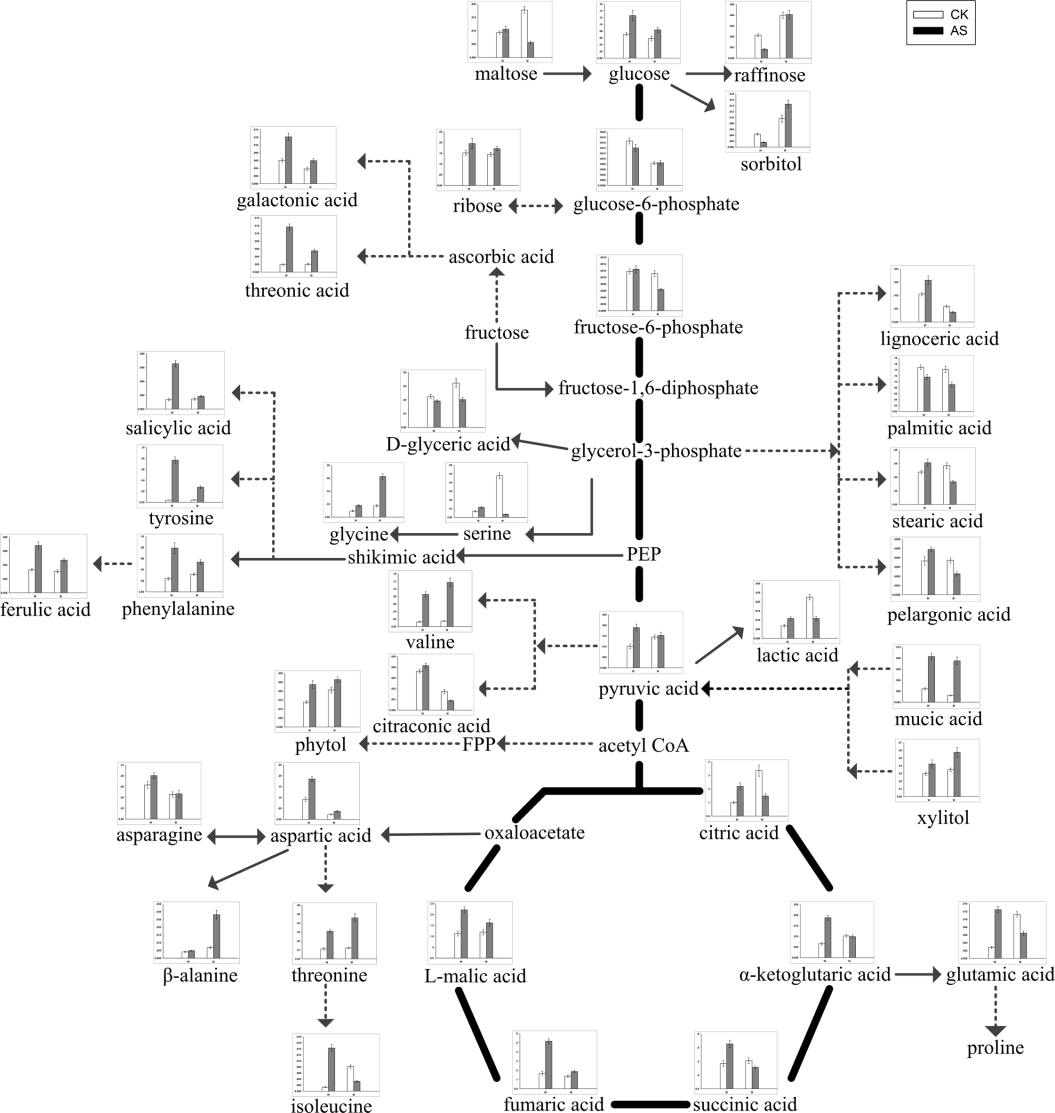
Changes in metabolites of the metabolic pathways in the leaves of the two soybean genotypes seedlings varied with salt tolerance 14 days after the imposition of alkali-salt stress. W and M on the X-axis indicate wild soybean and cultivated soybean, respectively. The values on the Y-axis indicate the relative concentration of metabolites. CK: control treatment, AS: alkali-salt stress.

Based on the metabolic responses of W and M under alkali-salt stress, 43 differential metabolites, including 8 carbohydrates and polyols, 10 organic acids, 11 amino acids, 5 fatty acids, and 9 metabolites associated with glycolysis and TCA, were screened and analyzed (Table 2). Most of carbohydrates and polyols, including ribose, phytol, xylitol and threitol, had similar responses and increased in both W and M under alkali-salt stress compared with in the control (Table 2). In addition, nine organic acids in W and six in M, under alkali-salt stress, increased compared with the control. The magnitudes of the increases in galactonic acid, threonic acid, ferulic acid and salicylic acid in W were obviously greater than in M. Furthermore, lactic acid, citraconic acid and citramalic acid in M, under alkali-salt stress, had lower levels than in the control (Table 2). Meanwhile, 11 amino acids in W increased under alkali-salt stress, and glycine, valine, threonine, aspartic acid, glutamic acid, phenylalanine and isoleucine increased significantly (P<0.05) in W. Most of the amino acids in M, including glycine, threonine, valine, aspartic acid, phenylalanine, asparagine, tyrosine and β-alanine, also accumulated (Table 2). A clear difference was observed in metabolite levels between W and M after two weeks of alkali-salt stress. Stearic acid, pelargonic acid and lignoceric acid accumulated in W under alkali-salt stress. However, stearic acid, palmitic acid, pelargonic acid and lignoceric acid decreased in M. The elevation of glucose, fructose-6-phosphate, pyruvic acid, citric acid, α-ketoglutaric acid, succinic acid, fumaric acid and L-malic acid in W induced by alkali-salt stress indicated that the processes of glycolysis and TCA are important for controlling alkali-salt stress-associated energy metabolism. However, the contents of citric acid, α-ketoglutaric acid and succinic acid were reduced in M, but glucose, glucose-6-phosphate and pyruvic acid were increased, while the accumulation levels of these metabolites were less than in W (Fig 5 and Table 2). In addition, the changes and differences in the levels of these metabolites in W and M seedlings under alkali-salt stress are shown in Fig 5.

**Table 2:** Relative concentrations and fold changes of differential metabolites in the leaves of W and M after 14 days of alkali-salt stress.

Metabolite name	Relative concentration					Fold changes	
	W		M		log_2_^(AS/CK)^		
	CK	AS	CK	AS	W		M
**Glycolysis and TCA**							
glucose	0.71±0.05	1.26±0.16	0.58±0.07	0.84±0.08	8.34**		5.31*
glucose-6-phosphate	0.02±0.00	0.01±0.00	0.01±0.00	0.01±0.00	-2.43		0.33
fructose-6-phosphate	0.01±0.00	0.01±0.00	0.01±0.00	0.01±0.00	0.81		-8.03*
pyruvic acid	0.10±0.01	0.19±0.02	0.14±0.01	0.15±0.02	9.06**		0.72
citric acid	0.99±0.07	2.19±0.27	3.35±0.42	1.48±0.16	11.47*		-11.76*
α-ketoglutaric acid	0.07±0.01	0.19±0.01	0.10±0.01	0.10±0.01	15.13*		-0.49
succinic acid	3.69±0.46	6.54±0.55	4.11±0.41	3.12±0.21	8.26**		-3.95*
fumaric acid	1.69±0.21	5.16±0.27	1.36±0.14	1.87±0.12	16.14**		4.52*
L-malic acid	11.19±1.12	22.10±1.47	11.93±1.33	16.18±1.62	9.82**		4.40*
**Amino acids**							
glycine	0.09±0.01	0.18±0.01	0.17±0.02	0.63±0.04	9.24**		18.49*
serine	0.07±0.01	0.11±0.01	0.48±0.03	0.04±0.00	7.13**		-37.40*
threonine	0.11±0.01	0.31±0.02	0.12±0.01	0.46±0.05	14.54**		18.97**
valine	0.13±0.01	0.85±0.07	0.15±0.01	1.17±0.12	27.66**		29.94*
aspartic acid	0.91±0.09	1.85±0.12	0.23±0.03	0.36±0.05	10.22**		6.87*
glutamic acid	0.03±0.00	0.12±0.01	0.11±0.01	0.06±0.01	21.49**		-8.08*
phenylalanine	0.24±0.02	0.79±0.10	0.32±0.02	0.53±0.04	17.28**		7.60*
asparagine	0.63±0.06	0.80±0.05	0.46±0.05	0.47±0.07	3.51		0.39
tyrosine	0.04±0.00	0.77±0.06	0.04±0.01	0.27±0.02	42.95**		27.55*
isoleucine	0.01±0.00	0.14±0.01	0.08±0.01	0.03±0.00	34.19**		-13.10*
β-alanine	0.04±0.00	0.05±0.00	0.07±0.01	0.28±0.03	2.44*		20.37**
**Carbohydrates and polyols**							
maltose	0.09±0.01	0.11±0.01	0.18±0.01	0.06±0.01	1.83		-16.66**
ribose	1.52±0.11	1.95±0.24	1.45±0.09	1.72±0.10	3.64*		2.43*
raffinose	0.02±0.00	0.01±0.00	0.04±0.00	0.04±0.00	-13.66*		0.24
levoglucosan	0.01±0.00	0.01±0.00	0.02±0.00	0.01±0.00	8.86*		-13.07**
phytol	0.14±0.01	0.24±0.02	0.21±0.02	0.26±0.01	7.72**		3.60*
xylitol	0.30±0.02	0.42±0.05	0.35±0.02	0.57±0.06	5.07*		7.22*
sorbitol	0.04±0.00	0.02±0.00	0.10±0.01	0.15±0.01	-14.25**		5.98*
threitol	0.01±0.00	0.03±0.00	0.01±0.00	0.02±0.00	16.97**		6.30*
**Fatty acids**							
stearic acid	0.48±0.03	0.61±0.06	0.57±0.04	0.33±0.02	3.66*		-7.75*
palmitic acid	1.49±0.08	1.16±0.09	1.42±0.11	0.91±0.08	-3.63**		-6.46**
1-monopalmitin	0.00±0.00	0.01±0.00	0.00±0.00	0.00±0.00	1.88*		-0.40
pelargonic acid	0.00±0.00	0.00±0.00	0.00±0.00	0.00±0.00	4.25*		-7.18**
lignoceric acid	0.02±0.00	0.03±0.00	0.01±0.00	0.01±0.00	5.76**		-6.62*
**Organic acids**							
galactonic acid	0.06±0.00	0.12±0.01	0.04±0.00	0.06±0.01	10.03*		6.38**
lactic acid	0.07±0.01	0.11±0.01	0.23±0.02	0.11±0.01	6.68**		-10.48*
threonic acid	0.02±0.00	0.12±0.01	0.02±0.00	0.05±0.00	25.90**		14.36**
mucic acid	0.02±0.00	0.08±0.01	0.01±0.00	0.08±0.01	17.55**		26.10**
citraconic acid	0.04±0.00	0.04±0.00	0.02±0.00	0.01±0.00	1.96*		-9.76**
salicylic acid	0.01±0.00	0.07±0.00	0.01±0.00	0.02±0.00	22.63*		3.45*
ferulic acid	0.03±0.00	0.07±0.01	0.03±0.00	0.05±0.00	10.47**		6.11*
3-cyanoalanine	0.10±0.01	0.14±0.01	0.08±0.01	0.09±0.01	5.17*		1.85
citramalic acid	0.01±0.00	0.01±0.00	0.02±0.00	0.01±0.00	1.42		-0.31
D-glyceric acid	0.23±0.01	0.19±0.01	0.32±0.03	0.20±0.01	-2.35*		-6.78*

The relative contents of metabolites are the mean of data from four biological replicates using GC-MS. The fold changes was calculated using the formula log_2_^(alkali-salt/control)^. Values were presented as the mean ±standard deviation of four biological replicates. Relative concentration values and standard deviation were increased 10 times in each treatment.

* and **indicate significant (P<0.05) and highly significant difference (P<0.01), respectively.

## Discussion

Plants produce osmotic stress and ion toxicity under salt stress, as well as a series of secondary effects, such as nutritional deficits and oxidative stress [28], and these stresses severely inhibit plant growth. In this study, M and W were subjected to neutral-salt and alkali-salt stresses, and the growth of the two genotypes were inhibited. The colors of the leaves and roots also changed correspondingly. The plant heights and root lengths were significantly decreased, especially in M. The absolute biomass and growth rate should also be taken into account when evaluating salt tolerance. The biomass accumulation and relative growth rate showed that the adaptability of W to both types of salt stress was obviously greater than that of M. At the same time, the degree of inhibition caused by salt stress on the roots was significantly higher than that on the above-ground plant parts, and the degree of inhibition under alkali-salt stress was significantly greater than that under neutral-salt stress. These results were consistent with the findings observed in our pervious study [24].

Plant salt tolerance is a special physiological and biochemical process in the growth and development of plants under salt stress. At present, the understanding of salt tolerance, including osmotic regulation, ion transport and balance, antioxidant protection, and late embryogenesis abundant proteins, is still limited [8]. To accommodate the osmotic balance between cytoplasm and environment, legumes accumulate low-molecular-weight metabolites, termed compatible solutes, which can contribute to reducing the water potential in the cytoplasm [29]. These compatible solutes include mainly organic acids, amino acids, carbohydrates and polyols [30]. Organic acid, as an osmotic adjuster of small molecules, plays an important role in the physiological process of salt resistance, especially under alkali-salt stress [31–33]. In the present study, most of the organic acids of the differential metabolites accumulated in W under neutral-salt stress, while the levels of organic acids decreased in M. Under the alkali salt stress, the contents of a majority of organic acids increased in W and M, but the accumulation in W was dramatically higher than that in M. This further underlines that the regulation of organic acid metabolism may be closely related to the salt tolerance mechanism of W. Salicylic acid, (SA) as a plant endogenous signal molecule, can induce or enhance the antioxidant system, remove the excess ROS in plants, and reduce the level of membrane lipid peroxidation in plant cells, to improve the metabolic activities of the cells and alleviate inhibition of salt stress on plant growth [34–37]. Additionally, it has important physiological functions in the resistance of plants to salt, drought, low temperature, disease and so on [38,39]. In this study, the SA content in W, but not in M, was increased under neutral-salt stress. Additionally, SA accumulated in both kinds of soybean, but the accumulation in W was obviously higher than in M under alkali-salt stress. Our study confirmed that the accumulation of SA was also an important mechanisms of salt tolerance in W. Free amino acids are not only the basic unit of protein synthesis, but also have a significant effect on plant resistance to stress [40]. Under neutral-salt stress, the two genotypes of soybean showed different responses, and the amino acid content, including glutamic acid, phenylalanine and glycine, was elevated in W, while the content in M decreased. The results indicated that W could increase the amino acid content to resist the neutral-salt stress. It is worth mentioning that the glutamate pathway is the main source of proline synthesis in the case of osmotic stress. At the same time, glutamic acid is the precursor of chlorophyll a, and chlorophyll b evolved from chlorophyll [41, 42]. The increase in the glutamic acid content in plants under salt stress may be related to photosynthesis [43]. In this study, glutamate levels in the leaves of W were increased in both salt stresses, while the glutamic acid content in M decreased, illustrating the essence of the difference. It is possible to synthesize more photosynthetic pigments by accumulating glutamic acid to increase the photosynthetic intensity, thereby, adapting to salt stress. Previous studies indicated that small molecular carbohydrates and polyols are the main osmotic adjustment substances in plants responding to salt stress [44]. However, in this test, the contents of maltose, raffinose and phytol were reduced, while the sorbitol content increased, in W, but the changes in the contents were opposite in M under the neutral-salt stress. Under the alkali-salt stress, the maltose and phytol contents increased, and the sorbitol and raffinose levels decreased in W. However, the contents of phytol, sorbitol and raffinose increased, while the maltose content was reduced in M. The small molecular carbohydrates and polyols in both genotypes showed no obvious regular changes, indicating that small molecular carbohydrates and polyols may not be the main osmotic adjustment substances of soybean resistance to salt stress.

Squalene is a naturally existing unsaturated triterpenoid connected by six isoprenes, and it has the function of carrying oxygen and antioxidants [45]. Squalene increases oxygen use in tissue cells by promoting metabolism and increases tolerance to hypoxia [46]. In addition, squalene can reduce intracellular ROS levels as shown by Warleta et al. [47] and also improves superoxide dismutase (SOD) activity. In this study, squalene was accumulated in both W and M under both kinds of stress, but the accumulation in W was greater than in M. The results suggest that W accumulated more squalene to remove excess ROS, improving the salt tolerance ability and, thereby, enhancing salt tolerance.

The contents of various plant metabolites were changed under salt stress, and some metabolic processes and metabolic pathways also adapted to the external environment. β-oxidation is the primary manner of fatty acid decomposition, which provides a large amount of energy needed for life activities, thus playing an important role in plant stress responses [48, 49]. Large amounts of fatty acids were accumulated in W under alkali-salt stress. This accumulation may prompt β-oxidation in W, generating large amounts of energy, which reduce the damage caused by a hostile environment. Whereas, the damage in M is so great under alkali-salt stress that energy production may suppressed. The contents of linoleic acid, stearic acid and palmitic acid in the two kinds of soybean under a neutral-salt treatment decreased, which may indicate that β-oxidation is not the main metabolic pathway in W when under a neutral-salt stress. Under a neutral-salt stress, the citric acid and α-ketoglutaric acid contents increased in W, and promoted the production of the TCA cycle. Compared with W, the neutral-salt stress forced M to reduce the citric acid and α-ketoglutaric acid contents significantly, so that the energy production process of TCA slowed down. Meanwhile, glycolysis, which may carry out anaerobic respiration, increased, resulting in the accumulation of products that produce toxic effects. Glycolysis and the TCA cycle were increased in W under alkali-salt stress, released more energy and accelerate the physiological metabolic reaction against stress. Under alkali-salt stress, the processes of glycolysis and the TCA cycle in M were similar to those under the neutral-salt stress. Under the two types of salt stresses, the processes of β-oxidation, glycolysis and the TCA cycle changed dramatically in W, which was advantageous to surviving in an adverse environment. This shows that W undergoes less damage in an external salt environment due to a variety of responses related to physiological metabolic plasticity, resulting in an improved salt tolerance.

## Conclusions

Based on the comparison of growth parameters and metabolic profiles between the two genotypes under alkali- and neutral-salt stresses, it may be concluded that the adaptability to salt stress and the salt tolerance mechanisms of W and M are very different. W has a higher content of compatible solutes, such as organic acids and amino acids, and a more active TCA cycle than M under neutral-salt stress. Additionally, W could induce the metabolic pathways responsible for energy generation, such as β-oxidation, glycolysis and the TCA cycle, to improve the alkali-salt tolerance over that found in M. Our study revealed that the salt-tolerant mechanisms in W were based on changes in the metabolic profiles. It has provided an important theoretical basis for the exploitation and effective use of soybean resources. Moreover, it has also provided an important reference for the improvement of salt tolerance and yields, as well as for developing new soybean cultivars.

## Supporting Information

S1 Table The growth parameters of wild soybean and cultivated soybean under normal and salt conditions. (DOCX)

S2 Table Differences of metabolite profiles in seedling leaves between wild soybean and cultivated soybean under neutral-salt and alkali-salt stress. (DOCX)

S3 Table The contribution of metabolites in seedling leaves to the first principal component (PC1) and the second principal component (PC2). (DOCX)

S4 Table Metabolite profiles changes in seedling leaves of wild soybean and cultivated soybean under normal condition. (DOCX)

## References

[1] Lam HM, Xu X, Liu X, Chen W, Yang G, Wong FL, et al Resequencing of 31 wild and cultivated soybean genomes identifies patterns of genetic diversity and selection. Nat Genet. 2010; 42: 1053–1059. 10.1038/ng.715 21076406

[2] Phang TH, Shao G, Lam HM. Salt tolerance in soybean. J Integ Plant Biol. 2008;50(10): 1196–1212. 10.1111/j.1744-7909.2008.00760.x 19017107

[3] Ledesma F, Lopez C, Ortiz D, Chen P, Korth KL, Ishibashi T, et al A simple greenhouse method for screening salt tolerance in soybean. Crop Sci. 2016; 56(2): 585–94.

[4] Ren SX, Weeda S, Li HW, Whitehead B, Guo YD, Atalay A, et al Salt tolerance in soybean WF-7 is partially regulated by ABA and ROS signaling and involves with holding toxic Cl^-^ ions from aerial tissues. Plant Cell Rep. 2012;31: 1527–1533. 10.1007/s00299-012-1268-2 22527198

[5] Zhu JK. Plant salt tolerance. Trends Plant Sci. 2001;6: 66–71. 11173290 10.1016/s1360-1385(00)01838-0

[6] Sanchez DH, Pieckenstain FL, Szymanski J, Erban A, Bromke M, Hannah MA, et al Comparative Functional Genomics of Salt Stress in Related Model and Cultivated Plants Identifies and Overcomes Limitations to Translational Genomics. PLoS One. 2011;6: 10.1371/journal.pone.0017094 PMC3038935 21347266

[7] Wang XP, Geng SJ, Ma YQ, Shi DC, Yang CW, Wang H. Growth, photosynthesis, solute accumulation, and ion balance of tomato plant under sodium- or potassium-salt stress and alkali stress. Agronomy J. 2015;107(2): 651–661.

[8] Lu YH, Lam HM, Pi E, Zhan QM, Tsai S, Wang CM, et al Comparative metabolomics in *glycine max* and *glycine soja* under salt stress to reveal the phenotypes of their offspring. J Agric Food Chem. 2013;61(36): 8711–21. 10.1021/jf402043m 23930713

[9] Xue ZC, Gao HY, Liu J. Different response of photosynthetic apparatus between wild soybean (*Glycine soja*) and cultivated soybean (*Glycine max*) to NaCl stress. Acta Ecologica Sinica. 2011;11: 3101–3109.

[10] Wei XY, Tang JX, Lu YZ. Effects of salt stress on germination of seed of different wild soybean. Seed. 2008;1: 68–70.

[11] Zhou S, Zhou M, Zhang S, Liu ZT, Zhao YJ, Yu TZ, et al Isoflavone soflavone accumulation in wild soybean under saline conditions and its ecological significance. J Plant Ecology. 2007;5: 930–936.

[12] Cai H, Zhu YM, Li Y, Bai X, Ji W, Wang DD, et al Solation and tolerance analysis of *GsNAC20* gene linked to response to stress in *Glycine soja*. ACTA Agron SINICA. 2011;8: 1351–1359.

[13] Chen XY. Plant secondary metabolism. World Sci-Tech R&D. 2006;5: 1–4.

[14] Fan XD, Wang JQ, Yang N, Dong YY, Liu L, Wang FW, et al Gene expression profiling of soybean leaves and roots under salt, saline-alkali and drought stress by high-throughput Illumina sequencing. Gene. 2013;512(2): 392–402. 10.1016/j.gene.2012.09.100 23063936

[15] Aghaei K, Ehsanpour AA, Shah AH, Komatsu S. Proteome analysis of soybean hypocotyl and root under salt stress. Amino Acids. 2009;36(1): 91–98. 10.1007/s00726-008-0036-7 18264660

[16] Zhang JT, Zhang Y, Du YY, Chen SY, Tang HR. Dynamic metabonomic responses of tobacco (*nicotiana tabacum*) plants to salt stress. J Proteome Res. 2011;10(4): 1904–14. 10.1021/pr101140n 21323351

[17] Wu DZ, Cai SG, Chen MX, Ye LZ, Chen ZH, Zhang HT, et al Tissue metabolic responses to salt stress in wild and cultivated barley. PLoS ONE. 2013;8(1): 10.1371/journal.pone.0055431 PMC3561194 23383190

[18] Sun CX, Li MQ, Gao XX, Liu LN, Wu XF, Zhou JH. Metabolic response of maize plants to multi-factorial abiotic stresses. Plant Biol. 2016;18: 120–9. 10.1111/plb.12305 25622534

[19] Guo R, Yang ZZ, Li F, Yan CR, Zhong XL, Liu Q, et al Comparative metabolic responses and adaptive strategies of wheat (*triticum aestivum*) to salt and alkali stress. BMC Plant Biol. 2015;15(1): 10.1186/s12870-015-0546-x PMC4492011 26149720

[20] Ampofo-Asiama J, Baiye VMM, Hertog MLATM, Waelkens E, Geeraerd AH, Nicolai BM. The metabolic response of cultured tomato cells to low oxygen stress. Plant Biol. 2014;16(3): 594–606. 10.1111/plb.12094 24119171

[21] Kim JK, Bamba T, Harada K, Fukusaki E, Kobayashi A. Time-coursemetabolic profiling in *Arabidopsis thaliana* cell cultures after salt stress treatment. J Exp Bot. 2007;58: 415–424. 17118972 10.1093/jxb/erl216

[22] Rizhsky L, Liang HJ, Shuman J, Shulaev V, Davletova S, Mittler R. When defense pathways collide the response of Arabidopsis to a combination of drought and heat stress. Plant Physiol. 2004;134: 1683–1696. 15047901 10.1104/pp.103.033431 PMC419842

[23] Shi LX, Ma S, Fang Y, Xu JY. Crucial variations in growth and ion homeostasis of *glycine gracilis* seedlings under two types of salt stresses. J Soil Sci Plant Nut. 2015;15: 1007–1023.

[24] Kingsbury RW, Epstein E, Peary RW. Physiological responses to salinity in selected lines of wheat. Plant Physio. 1984;74: 417–423. 10.1104/pp.74.2.417 PMC1066693 16663433

[25] Allwood JW, Erban A, Koning SD, Dunn WB, Luedemann A, Lommen A, et al Inter-laboratory reproducibility of fast gas chromatography-electron impact-time of flight mass spectrometry (GC-EI-TOF/MS) based plant metabolomics. Metabolomics. 2009;5: 479–496. 20376177 10.1007/s11306-009-0169-z PMC2847149

[26] Kind T, Wohlgemuth G, Lee DY, Lu Y, Palazoglu M, Shahbaz S, et al FiehnLib: Mass spectral and retention index libraries for metabolomics based on quadrupole and time-of-flight gas chromatography/mass spectrometry. Anal Chem. 2009;81: 10038–10048. 10.1021/ac9019522 19928838 PMC2805091

[27] Xia J, Mandal R, Sinelnikov I, Broadhurst D, Wishart DS. MetaboAnalyst 2.0-a comprehensive server for metabolomic data analysis. Nucl Acids Res. 2012;40: 127–133. 10.1093/nar/gks374 PMC3394314 22553367

[28] Zhu JK. Regulation of ion homeostasis under salt stress. Curr Opin PlantBiol. 2003;6: 441–445. 10.1016/s1369-5266(03)00085-2 12972044

[29] Phang TH, Shao GH, Lam HM. Salt tolerance in soybean. J Integr Plant Biol. 2008;50: 1196–1212. 10.1111/j.1744-7909.2008.00760.x 19017107

[30] Zhang HY, Zhao KF. Effects of salt and water stresses on osmotic adjustment of *Suaeda salsa* seedlings. Acta Bot Sin. 1998;40(1): 56–61.

[31] Shi DC, Sheng YM. Effect of various salt-alkaline mixed stress conditions on sunflower seedling and analysis of their stress factors. Environ J Exp Bot. 2005;54: 8–21.

[32] Shi DC, Yin SJ, Yang GH, Zhao KY. Citric acid accumulation in an alkali-tolerant plant *Puccinellia tenuiflora* under alkaline stress. Acta Bot Sin. 2002;44(5): 537–540.

[33] Yang CW, Chong JN, Kim CM, Li CY, Shi DC, Wang DL. Osmotic adjustment and ion balance traits of an alkali resistant halophyte *Kochia sieversiana* during adaptation to salt and alkali conditions. Plant Soil. 2007;294: 263–276.

[34] Gunes A, Inal A, Alpaslan M, Cicek N, Guneri E, Eraslan F, et al Effects of exogenously applied salicylic acid on the induction of multiple stress tolerance and mineral nutrition in maize (*Zea mays* L.). Arch Agron Soil Science. 2005;51(6): 687–695.

[35] Stevens J, Senaratna T, Sivasithamparam K. Salicylic Acid Induces Salinity Tolerance in Tomato (*Lycopersicon esculentum* cv. Roma): Associated Changes in Gas Exchange, Water Relations and Membrane Stabilisation. Plant Growth Regul. 2006;49(1): 77–83.

[36] Bai T, Li CY, Ma FW, Shu HR, Han MY. Exogenous Salicylic Acid Alleviates Growth Inhibition and Oxidative Stress Induced by Hypoxia Stress in *Malus robusta* Rehd. Plant Growth Regul. 2009;28(4): 358–366.

[37] Yusuf M, Hasan SA, Ali B, Hayat S, Fariduddin Q, Ahmad A. Effect of Salicylic Acid on Salinity-induced Changes in *Brassica juncea*. J Integr Plant Bio. 2008;50(9): 1096–1102. 18844778 10.1111/j.1744-7909.2008.00697.x

[38] Jiang ZS, Chen XW. Effect of Salicylic Acid on Drought Resistance of Three Rinds of Shrub Seedlings. J Soil Water Conser. 2004;18(2): 166–169.

[39] Zhang SG, Gao JY, Song JZ. Effect of salicylic acid on salt-resistance of wheat (*Triticun aestivum* L.). Chin J Appl & Envir Bio. 1999;5(3): 264–267.

[40] Less H, Galili G. Principal transcriptional programs regulating plant amino acid metabolism in response to abiotic stresses. Plant Physiol. 2008;147: 316–330. 10.1104/pp.108.115733 18375600 PMC2330312

[41] Woodward RB, Ayer WA, Beaton JM, Bickelhaupt F, Bonnett R, Buchschacher P, et al The total synthesis of chlorophyll a. Tetrahedron. 1990;46(22): 7599–7659.

[42] Ito H, Takaichi S, Tsuji H, Tanaka A. Properties of synthesis of chlorophyll a from chlorophyll b in cucumber etioplasts. J Bio Chem. 1994;269(35): 22034–22038. 8071325

[43] Gong B, Wen D, VandenLangenberg K, Wei M, Yang FJ, Shi QH, et al Comparative effects of NaCl and NaHCO_3_ stress on photosynthetic parameters, nutrient metabolism, and the antioxidant system in tomato leaves. Sci Hortic. 2013;157: 1–12.

[44] Sheen J, Zhou L, Jang JC. Sugars as signaling molecules. Curr Opin Plant Biol. 1999;2(5): 410–418. 10508760 10.1016/s1369-5266(99)00014-x

[45] Chen XB, Shi XM, Zhao ZN, Zhou XJ, Cao YP, Jiang YM. Progress of squalene extraction from vegetable oils. Chin Oils and Fats. 2013;11: 72–75.

[46] Liu CY, Ma MH, Jin GF, Geng F, Wang QL, Sun SG. Research progress of the biological activity squalene. J Chin Instit Food Sci and Tech. 2015;5: 147–156.

[47] Warleta F, Campos M, Yosra A, Sanchez-Quesada C, Ruiz-Mora J, Beltran G. Squalene protects against oxidative DNA damage in MCF10A human mammary epithelial cells but not in MCF7 and MDA-MB-231 human breast cancer cells. Food and Chem Toxico. 2010;48(4): 1092–1100. 10.1016/j.fct.2010.01.031 20138105

[48] Xu J, Li LH, Zhu JH, Peng SQ. Insights into Fatty Acids β-Oxidation in Plant. Letters in Biotech. 2008;1: 141–144.

[49] Cooper TG, Beevers H. β- oxidation in glyoxysomes from castor bean endosperm. J Biol Chem. 1969; 244: 3514–3520. 4307455

