# Supplementary material for: Metabolic Profiles Reveal Changes in Wild and Cultivated Soybean Seedling Leaves under Salt Stress
Source: PLoS One. 2016 Jul 21;11(7):e0159622. doi: 10.1371/journal.pone.0159622 (PMC4956222; doi:10.1371/journal.pone.0159622)
Supplement: S1 Table — (DOCX) [file pone.0159622.s001.docx]

**S1 Table. The growth parameters of wild soybean and cultivated soybean under normal and salt conditions.**

| Growth parameter | log_2_^(treatment/control)^ | | | | | | | |
| --- | --- | --- | --- | --- | --- | --- | --- | --- |
|  | W(NS/CK) | | W(AS/CK) | | M(NS/CK) | | M(AS/CK) | |
| Shoot height (cm) | -0.28 | P<0.05 | -0.60 | P<0.05 | -0.36 | NS | -0.68 | P<0.05 |
| Root length (cm) | -0.18 | P<0.05 | -0.29 | NS | -0.21 | P<0.05 | -0.30 | P<0.05 |
| Dry weight of shoots (g) | -0.03 | NS | -0.23 | P<0.05 | -0.03 | P<0.05 | -0.20 | P<0.05 |
| Dry weight of roots (g) | -0.75 | P<0.05 | -1.05 | NS | -0.30 | NS | -0.64 | NS |
| RGR of shoots | -0.26 | P<0.05 | -0.58 | P<0.05 | -0.42 | NS | -0.42 | NS |
| RGR of roots | -0.32 | P<0.05 | -0.32 | NS | -1.00 | P<0.05 | -1.00 | P<0.05 |

RGR were calculated using the formula (In DW_1_ – In DW_0_) / t_2_ – t_1_.The fold changes were calculated using the formula log_2_^(treatment/control)^. P<0.05 means significant difference; NS means non-significant difference.
