# Supplementary material for: Metabolic Profiles Reveal Changes in Wild and Cultivated Soybean Seedling Leaves under Salt Stress
Source: PLoS One. 2016 Jul 21;11(7):e0159622. doi: 10.1371/journal.pone.0159622 (PMC4956222; doi:10.1371/journal.pone.0159622)
Supplement: S2 Table — (DOCX) [file pone.0159622.s002.docx]

**S2 Table. Differences of metabolite profiles in seedling leaves between wild soybean and cultivated soybean under neutral-salt and alkali-salt stress.**

| metabolite name | log_2_^(treatment/control)^ | | | | | | | |
| --- | --- | --- | --- | --- | --- | --- | --- | --- |
|  | W(NS/CK) | | W(AS/CK) | | M(NS/CK) | | M(AS/CK) | |
| alanine | -0.24 | P<0.05 | -0.88 | P<0.05 | -1.05 | P<0.01 | -1.07 | P<0.05 |
| glycine | 0.39 | P<0.05 | -0.51 | P<0.05 | 0.92 | P<0.01 | 1.85 | P<0.05 |
| serine | 1.38 | P<0.05 | -0.68 | P<0.05 | 0.71 | P<0.01 | -3.74 | P<0.05 |
| threonine | 0.37 | P<0.05 | -0.16 | NS | 1.45 | P<0.01 | 1.90 | P<0.01 |
| valine | 0.07 | NS | 0.04 | NS | 2.77 | P<0.01 | 2.99 | P<0.05 |
| aspartic acid | -0.32 | P<0.05 | -0.74 | P<0.05 | 1.02 | P<0.01 | 0.69 | P<0.05 |
| glutamic acid | 1.97 | P<0.01 | -1.19 | P<0.05 | 2.15 | P<0.01 | -0.81 | P<0.05 |
| phenylalanine | 0.49 | P<0.05 | -1.97 | P<0.01 | 1.73 | P<0.01 | 0.76 | P<0.05 |
| asparagine | 1.21 | P<0.05 | -2.04 | P<0.01 | 0.35 | NS | 0.04 | NS |
| tyrosine | 1.41 | P<0.05 | -0.89 | P<0.05 | 4.30 | P<0.01 | 2.75 | P<0.05 |
| isoleucine | 1.46 | P<0.05 | -0.73 | P<0.05 | 3.42 | P<0.01 | -1.31 | P<0.05 |
| β-alanine | -0.56 | P<0.05 | -0.28 | NS | 0.24 | P<0.05 | 2.04 | P<0.01 |
| maltose | -0.38 | NS | 0.17 | NS | 0.18 | P<0.05 | -1.67 | P<0.01 |
| glucose | -0.74 | P<0.05 | 0.09 | NS | 0.83 | P<0.01 | 0.53 | P<0.05 |
| ribose | -0.45 | P<0.05 | -0.46 | P<0.05 | 0.36 | P<0.05 | 0.24 | P<0.05 |
| raffinose | -0.05 | NS | 0.27 | P<0.05 | -1.37 | P<0.05 | 0.02 | NS |
| levoglucosan | 0.68 | P<0.05 | -1.02 | P<0.05 | 0.89 | P<0.05 | -1.31 | P<0.01 |
| phytol | -0.25 | P<0.05 | 0.13 | P<0.05 | 0.77 | P<0.01 | 0.36 | P<0.05 |
| xylitol | -0.04 | NS | -0.21 | P<0.05 | 0.51 | P<0.05 | 0.72 | P<0.05 |
| sorbitol | 2.32 | P<0.05 | -0.68 | P<0.05 | -1.42 | P<0.01 | 0.60 | P<0.05 |
| threitol | 0.61 | P<0.05 | -0.78 | P<0.05 | 1.70 | P<0.01 | 0.63 | P<0.05 |
| glucose-6-phosphate | -0.49 | P<0.05 | 1.08 | P<0.05 | -0.24 | NS | 0.03 | NS |
| fructose-6-phosphate | -0.47 | P<0.05 | 0.84 | P<0.05 | 0.08 | NS | -0.80 | P<0.05 |
| glycerol | -0.23 | P<0.05 | -0.02 | NS | -0.12 | P<0.05 | 0.21 | P<0.05 |
| linolenic acid | -1.05 | P<0.05 | 0.07 | P<0.05 | -0.37 | P<0.05 | -0.26 | P<0.05 |
| linoleic acid | -0.86 | P<0.05 | -0.14 | P<0.05 | -0.20 | NS | 0.08 | NS |
| stearic acid | -0.35 | P<0.05 | -0.04 | P<0.05 | 0.37 | P<0.05 | -0.78 | P<0.05 |
| palmitic acid | -0.34 | P<0.05 | -0.02 | P<0.05 | -0.36 | P<0.01 | -0.65 | P<0.01 |
| pelargonic acid | 0.13 | P<0.05 | -0.49 | P<0.01 | 0.43 | P<0.05 | -0.72 | P<0.01 |
| lignoceric acid | -0.09 | P<0.05 | 0.49 | P<0.05 | 0.58 | P<0.01 | -0.66 | P<0.05 |
| 4-hydroxycinnamic acid | -1.06 | P<0.05 | 0.78 | P<0.01 | 0.46 | P<0.05 | 0.68 | P<0.01 |
| 1-monopalmitin | -0.26 | P<0.05 | -0.41 | P<0.05 | 0.19 | P<0.05 | -0.04 | NS |
| 4-hydroxybutyrate | -0.63 | P<0.05 | 0.52 | P<0.05 | -0.23 | P<0.05 | 0.10 | NS |
| fumaric acid | -0.44 | P<0.05 | 0.26 | NS | 1.61 | P<0.01 | 0.45 | P<0.05 |
| galactonic acid | -0.58 | P<0.05 | -0.20 | P<0.05 | 1.00 | P<0.05 | 0.64 | P<0.01 |
| lactic acid | 0.24 | P<0.05 | 0.48 | P<0.05 | 0.67 | P<0.01 | -1.05 | P<0.05 |
| succinic acid | -0.16 | P<0.05 | 0.26 | P<0.05 | 0.83 | P<0.01 | -0.40 | P<0.05 |
| benzoic acid | 0.12 | P<0.05 | -0.62 | P<0.05 | 0.52 | P<0.05 | 0.11 | NS |
| pyruvic acid | 0.05 | NS | 0.34 | NS | 0.91 | P<0.01 | 0.07 | NS |
| threonic acid | 0.18 | P<0.05 | -0.58 | P<0.05 | 2.59 | P<0.01 | 1.44 | P<0.01 |
| mucic acid | -1.10 | P<0.05 | 0.81 | P<0.05 | 1.76 | P<0.01 | 2.61 | P<0.01 |
| oxoproline | 1.22 | P<0.05 | -0.70 | P<0.05 | 1.39 | P<0.01 | -0.96 | P<0.05 |
| glycolic acid | -0.04 | NS | -0.71 | P<0.05 | 0.83 | P<0.01 | -0.83 | P<0.01 |
| 4-aminobutyric acid | -0.31 | P<0.05 | -0.15 | P<0.05 | -0.37 | P<0.05 | -0.10 | P<0.05 |
| citraconic acid | 0.01 | NS | -0.55 | P<0.01 | 0.20 | P<0.05 | -0.98 | P<0.01 |
| salicylic acid | 0.68 | P<0.05 | -0.96 | P<0.05 | 2.26 | P<0.05 | 0.34 | P<0.05 |
| α-ketoglutaric acid | 0.94 | P<0.05 | -1.33 | P<0.01 | 1.51 | P<0.05 | -0.05 | NS |
| proline | -2.82 | P<0.05 | -0.79 | P<0.05 | 0.52 | P<0.05 | 5.80 | P<0.01 |
| citric acid | 1.02 | P<0.05 | -1.55 | P<0.01 | 1.15 | P<0.05 | -1.18 | P<0.05 |
| dehydroascorbic acid | 0.00 | NS | 0.31 | P<0.05 | -0.58 | P<0.05 | -0.34 | P<0.05 |
| L-malic acid | 0.28 | P<0.05 | -0.47 | P<0.05 | 0.98 | P<0.01 | 0.44 | P<0.05 |
| ferulic acid | -0.61 | P<0.05 | -0.33 | P<0.05 | 1.05 | P<0.01 | 0.61 | P<0.05 |
| 6-phosphogluconic acid | -0.48 | P<0.05 | 0.00 | NS | 0.16 | NS | 0.80 | P<0.01 |
| 2-ketoadipate | 0.13 | NS | -1.54 | P<0.01 | 1.38 | P<0.01 | -0.06 | NS |
| 3-hydroxypropionic acid | -0.27 | P<0.05 | 0.06 | NS | -0.27 | P<0.05 | -0.44 | P<0.01 |
| methylmalonic acid | 0.07 | NS | -0.49 | P<0.05 | 0.93 | P<0.01 | 1.93 | P<0.01 |
| 3-cyanoalanine | 1.41 | P<0.05 | -1.28 | P<0.05 | 0.52 | P<0.05 | 0.19 | NS |
| citramalic acid | 0.16 | P<0.05 | -0.74 | P<0.01 | 0.14 | NS | -0.03 | NS |
| D-glyceric acid | -0.07 | NS | -0.05 | NS | -0.24 | P<0.05 | -0.68 | P<0.05 |
| D-(glycerol 1-phosphate) | -0.57 | P<0.05 | -0.43 | P<0.05 | -0.03 | NS | 1.38 | P<0.01 |
| squalene | 0.99 | P<0.01 | 0.60 | P<0.05 | 1.52 | P<0.01 | 0.35 | P<0.05 |
| ethanolamine | -0.30 | P<0.05 | -0.52 | P<0.01 | -0.10 | P<0.05 | 0.11 | P<0.05 |
| N-acetyl-β-D-mannosamine | 0.48 | P<0.05 | -0.80 | P<0.01 | 1.13 | P<0.01 | -0.59 | P<0.01 |
| N-acetyl-D-galactosamine | 0.77 | P<0.05 | -0.80 | P<0.01 | -0.42 | P<0.05 | -0.68 | P<0.01 |
| maleimide | 0.75 | P<0.05 | -0.58 | P<0.05 | 0.21 | P<0.05 | -0.48 | P<0.05 |
| methyl phosphate | -0.71 | P<0.05 | 0.09 | NS | 0.52 | P<0.05 | 0.65 | P<0.05 |
| uracil | -0.47 | P<0.05 | -0.47 | P<0.05 | -0.93 | P<0.01 | -0.15 | P<0.05 |
| 2-hydroxypyridine | 0.01 | NS | -0.04 | NS | 0.99 | P<0.01 | 0.10 | P<0.05 |

The fold changes were calculated using the formula log_2_^(treatment/control)^. P<0.05 and P<0.01 mean significant and highly significant difference, respectively; NS means non-significant difference.
