# Supplementary material for: Metabolic Profiles Reveal Changes in Wild and Cultivated Soybean Seedling Leaves under Salt Stress
Source: PLoS One. 2016 Jul 21;11(7):e0159622. doi: 10.1371/journal.pone.0159622 (PMC4956222; doi:10.1371/journal.pone.0159622)
Supplement: S3 Table — (DOCX) [file pone.0159622.s003.docx]

**S3 Table. The contribution of metabolites in seedling leaves to the first principal component (PC1) and the second principal component (PC2).**

| metabolite name | PC1 | PC2 |
| --- | --- | --- |
| 2-hydroxypyridine | 0.00 | 0.15 |
| pyruvic acid | -0.04 | 0.18 |
| lactic acid | -0.09 | 0.07 |
| glycolic acid | -0.02 | 0.10 |
| maleimide | 0.14 | -0.04 |
| alanine | 0.02 | 0.04 |
| 3-hydroxypropionic acid | 0.02 | -0.03 |
| methyl phosphate | 0.06 | 0.16 |
| isoleucine | 0.02 | 0.10 |
| methylmalonic acid | 0.09 | 0.05 |
| valine | 0.17 | 0.08 |
| 4-hydroxybutyrate | -0.03 | 0.12 |
| benzoic acid | 0.01 | 0.05 |
| serine | -0.10 | 0.03 |
| ethanolamine | 0.04 | 0.11 |
| glycerol | 0.01 | 0.07 |
| proline | 0.18 | 0.10 |
| glycine | 0.05 | 0.10 |
| succinic acid | 0.00 | 0.11 |
| D-glyceric acid | -0.05 | 0.12 |
| uracil | 0.11 | -0.03 |
| citraconic acid | 0.04 | -0.01 |
| fumaric acid | 0.07 | 0.19 |
| pelargonic acid | 0.02 | 0.02 |
| 3-cyanoalanine | 0.36 | -0.15 |
| threonine | 0.19 | 0.08 |
| β-alanine | 0.07 | 0.15 |
| citramalic acid | -0.01 | 0.10 |
| L-malic acid | 0.06 | 0.11 |
| threitol | 0.02 | 0.01 |
| asparagine | 0.38 | -0.15 |
| salicylic acid | 0.12 | 0.14 |
| aspartic acid | 0.39 | -0.09 |
| oxoproline | 0.08 | 0.04 |
| 4-aminobutyric acid | 0.02 | 0.11 |
| threonic acid | 0.10 | 0.18 |
| α-ketoglutaric acid | 0.03 | 0.08 |
| glutamic acid | 0.35 | -0.10 |
| phenylalanine | 0.38 | -0.09 |
| 2-ketoadipate | 0.09 | 0.16 |
| ribose | 0.05 | 0.20 |
| xylitol | 0.03 | 0.14 |
| levoglucosan | -0.02 | 0.06 |
| D-(glycerol 1-phosphate) | 0.07 | 0.15 |
| citric acid | 0.03 | 0.01 |
| dehydroascorbic acid | -0.06 | 0.09 |
| glucose | 0.04 | 0.16 |
| sorbitol | -0.03 | -0.07 |
| tyrosine | 0.16 | 0.10 |
| 4-hydroxycinnamic acid | 0.03 | 0.12 |
| galactonic acid | 0.06 | 0.16 |
| mucic acid | 0.11 | 0.15 |
| palmitic acid | 0.00 | 0.03 |
| N-acetyl-D-galactosamine | 0.01 | -0.06 |
| ferulic acid | 0.07 | 0.16 |
| N-acetyl-β-D-mannosamine | 0.03 | 0.12 |
| phytol | 0.01 | 0.16 |
| linoleic acid | 0.03 | 0.11 |
| linolenic acid | 0.00 | 0.12 |
| stearic acid | 0.01 | 0.09 |
| fructose-6-phosphate | 0.04 | 0.08 |
| glucose-6-phosphate | 0.03 | 0.05 |
| 6-phosphogluconic acid | 0.08 | 0.14 |
| 1-monopalmitin | 0.01 | 0.08 |
| maltose | -0.08 | 0.18 |
| squalene | -0.02 | 0.11 |
| lignoceric acid | 0.02 | 0.05 |
| raffinose | 0.00 | 0.41 |
