# Supplementary material for: Metabolic Profiles Reveal Changes in Wild and Cultivated Soybean Seedling Leaves under Salt Stress
Source: PLoS One. 2016 Jul 21;11(7):e0159622. doi: 10.1371/journal.pone.0159622 (PMC4956222; doi:10.1371/journal.pone.0159622)
Supplement: S4 Table — (DOCX) [file pone.0159622.s004.docx]

**S4 Table. Metabolite profiles changes in seedling leaves of wild soybean and cultivated soybean under normal condition**

| metabolite name | log_2_^(W/M)^ | P |
| --- | --- | --- |
|  |  |  |
| pelargonic acid | -9.85 | P<0.01 |
| serine | -2.78 | P<0.05 |
| isoleucine | -2.61 | P<0.01 |
| glutamic acid | -2.01 | P<0.01 |
| lactic acid | -1.72 | P<0.01 |
| levoglucosan | -1.58 | P<0.01 |
| oxoproline | -1.50 | P<0.01 |
| N-acetyl-D-galactosamine | -1.25 | P<0.01 |
| glycolic acid | -1.24 | P<0.01 |
| sorbitol | -1.13 | P<0.01 |
| alanine | -1.01 | P<0.05 |
| benzoic acid | -1.00 | P<0.01 |
| maltose | -0.93 | P<0.01 |
| raffinose | -0.91 | P<0.05 |
| glycine | -0.88 | P<0.01 |
| 2-ketoadipate | -0.83 | P<0.05 |
| β-alanine | -0.74 | P<0.05 |
| citramalic acid | -0.68 | P<0.01 |
| 2-hydroxypyridine | -0.65 | P<0.01 |
| threitol | -0.65 | P<0.01 |
| α-ketoglutaric acid | -0.64 | P<0.05 |
| squalene | -0.61 | P<0.05 |
| phytol | -0.57 | P<0.05 |
| N-acetyl-β-D-mannosamine | -0.52 | P<0.05 |
| pyruvic acid | -0.52 | P<0.05 |
| D-glyceric acid | -0.52 | P<0.05 |
| phenylalanine | -0.40 | P<0.05 |
| stearic acid | -0.26 | P<0.05 |
| D-(glycerol 1-phosphate) | -0.23 | P<0.05 |
| valine | -0.23 | NS |
| xylitol | -0.23 | P<0.05 |
| linolenic acid | -0.18 | P<0.05 |
| succinic acid | -0.15 | P<0.05 |
| threonine | -0.13 | NS |
| L-malic acid | -0.09 | NS |
| salicylic acid | -0.09 | NS |
| ethanolamine | -0.09 | P<0.05 |
| threonic acid | -0.06 | NS |
| tyrosine | -0.06 | NS |
| 4-hydroxybutyrate | 0.01 | NS |
| 1-monopalmitin | 0.02 | NS |
| maleimide | 0.02 | NS |
| ribose | 0.06 | NS |
| palmitic acid | 0.07 | NS |
| fructose-6-phosphate | 0.08 | NS |
| ferulic acid | 0.11 | NS |
| methyl phosphate | 0.15 | NS |
| 6-phosphogluconic acid | 0.16 | NS |
| glycerol | 0.22 | P<0.01 |
| 4-aminobutyric acid | 0.25 | P<0.05 |
| linoleic acid | 0.27 | P<0.05 |
| 3-cyanoalanine | 0.27 | NS |
| glucose | 0.29 | P<0.05 |
| dehydroascorbic acid | 0.30 | P<0.05 |
| fumaric acid | 0.31 | NS |
| asparagine | 0.47 | P<0.05 |
| uracil | 0.59 | P<0.01 |
| galactonic acid | 0.66 | P<0.05 |
| 3-hydroxypropionic acid | 0.83 | P<0.05 |
| lignoceric acid | 0.84 | P<0.01 |
| mucic acid | 1.00 | P<0.05 |
| glucose-6-phosphate | 1.00 | P<0.05 |
| citraconic acid | 1.06 | P<0.01 |
| 4-hydroxycinnamic acid | 1.25 | P<0.01 |
| proline | 1.58 | P<0.05 |
| methylmalonic acid | 1.70 | P<0.01 |
| aspartic acid | 2.01 | P<0.01 |
| citric acid | 8.06 | P<0.05 |

The fold changes were calculated using the formula log_2_^(W/M)^. P<0.05 and P<0.01 mean significant and highly significant difference, respectively; NS means non-significant difference.
